# Views of People With Epilepsy About Web-Based Self-Presentation: A Qualitative Study

**DOI:** 10.2196/10349

**Published:** 2018-12-21

**Authors:** Alison Ruth McKinlay, Leone Lorna Ridsdale

**Affiliations:** 1 Department of Basic & Clinical Neuroscience Institute of Psychiatry, Psychology & Neuroscience King's College London London United Kingdom

**Keywords:** epilepsy, internet, social stigma, social media, eHealth, social networking, social support, self-management

## Abstract

**Background:**

Web-based media, particularly social networking sites (SNSs), are a source of support for people with long-term conditions, like epilepsy. Living with epilepsy can reduce opportunities for accessing information and social support owing to transportation difficulties and stigma leading to self-isolation. However, some people with epilepsy (PWE) overcome these barriers using SNSs and other Web-based media. At present, little is known about Web-based identity and self-presentation of PWE; this study aims to address this gap.

**Objective:**

This study aims to describe how the use of digital technologies, such as SNSs, impacts sense of identity in PWE.

**Methods:**

We used qualitative research methods to examine Web-based media use and self-presentation in a group of 14 PWE (age range: 33-73 years; 7 men and 7 women). The median diagnosis duration was 25 years. Semistructured interviews ranged from 40 to 120 minutes, held at participants’ homes or in a public place of their choice, in the United Kingdom. QSR Nvivo 11 software was used to perform an inductive thematic analysis.

**Results:**

In this study, 9 participants used Web-based media to “silently” learn from other PWE by reading user posts on SNSs and epilepsy-related forums. When asked about self-presentation, 7 participants described feeling cautious about disclosing their epilepsy to others online. Six participants presented themselves in the same manner irrespective of the situation and described their identity as being presented in the same way both online and offline.

**Conclusions:**

PWE can deploy SNSs and Web-based media to manage aspects of their condition by learning from others and obtaining social support that may otherwise be difficult to access. Some PWE share openly, whereas others silently observe, without posting. Both benefit from the shared experiences of others. Privacy concerns and stigma can act as a barrier to sharing using Web-based media and SNSs. For some, Web-based media offers a chance to experiment with identity and change self-presentation, leading to gradually “coming out” and feeling more comfortable discussing epilepsy with others.

## Introduction

Web-based media, including mobile phone apps and social networking sites (SNSs), enable the presentation of a *curated self*, where users can share selected aspects of their lives such as new relationships, pictures, and personal insights [1]. In June 2017, it was estimated that the number of monthly Facebook users had reached 2 billion [2] with 90% of older adults having used this SNS to access and share health-related information [3]. The phenomenon of using Web-based media as a health care aid is being referred to as *Health 2.0* [4]. Users can share their experiences of illness and learn about health care self-management in a Web-based environment [5]. Some users may even modify their offline health care with information gathered online.

Web-based media may be used in place of a traditional face-to-face support group, with similar or added benefits, including the possibility to remain anonymous [6]. Anonymity may protect users against social stigma [6], where an individual is thought to have characteristics perceived as undesirable or unacceptable [7]. Through Web-based media, users can access information and support in real time that people with health conditions might have otherwise waited for a medical consultation to access. In one study of people with diabetes, Facebook groups were used to share experiences and gain knowledge through the experiences of others [8]. People with other long-term conditions may gain similar benefits from Web-based media use [9].

As a result of increased SNS use, where users can disclose information about themselves to others [10,11], topics such as Web-based identity and self-presentation have become a focus of research [12]. In 1959, Goffman described identity presentation as actors performing on stage; different aspects of an individual’s identity are presented in different contexts [13]; this now also occurs in Web-based spaces [14]. SNSs provide a platform for identity performance, where practices in self-presentation can undergo continued maintenance and revision [15]. When Web-based users modify identity portrayal to fit with what is perceived to be an *ideal self*, these performances can be curated and reused in other social contexts [12,16].

Self-presentation is defined as the deliberate act of portraying identity in a certain, premeditated manner [17]. Practices in self-presentation, including deliberate nondisclosure, can mask users’ daily struggles [18], creating bias and even misrepresentation of experiences shared online. In addition, self-presentation can be influenced by stigma, which has been associated with long-term health conditions such as psychosis, epilepsy, and HIV or AIDS [19]. Through the use of online support groups and user forums, people with stigmatized health conditions can share the aspects of their identity with others and may feel safer to do so than face-to-face interactions [6]. Past research has focused on the impact of Web-based self-presentation on long-term conditions, such as diabetes, chronic fatigue [20], and chronic cough [21], but a gap exists in the literature on epilepsy and Web-based identity.

Epilepsy is a neurological condition characterized by recurrent seizures, affecting 1% of the United Kingdom (UK) population [22] and approximately 50 million people worldwide [23]. Treatment consists of medication use and advice on self-management. Despite treatment, up to 40% of people continue to experience seizures [23] and low quality of life [24]. People with epilepsy (PWE) frequently use the internet for information about epilepsy [25], and the wider literature show the benefit of Web-based communities in the self-management of other long-term conditions [9]. The benefits of the SNS use may be especially helpful for PWE, where unpredictability of seizures, transportation difficulty owing to a lack of driving license, and stigma, can limit access to face-to-face sources of information and support. This research aims to describe the Web-based practices of PWE and explore how SNSs use is associated with identity and self-presentation.

## Methods

### Study Design

We used a qualitative, cross-sectional research design, using purposive sampling methods and semistructured interviews. Ethical approval was granted by the Psychiatry, Nursing and Midwifery Research Ethics subcommittee at King’s College London (PNM/13/14-18). All participants were given an information sheet and gave informed consent at the start of their interview.

### Participants and Recruitment

Researchers advertised for volunteers through the webpage of the largest official UK epilepsy user group (Epilepsy Action, hereafter referred to as “user-group”), as well as their email list of 3500 members. The study eligibility criteria included the following: aged ≥17 years; self-reported epilepsy diagnosis; and experience of accessing user-group website information.

Overall, 35 PWE emailed the research team to register their interest in participating. Fifteen people attended interviews, with the final sample size of 14; this number of participants is acceptable in qualitative research [26], where the aim is to describe and explore rather than generalize. Of 20 who did not attend interviews, 2 respondents were based remotely and unable to attend interviews in-person, 2 respondents canceled their appointments owing to family emergency or holiday, and the remainder did not respond to further interview invitations. No incentives were offered to any participant involved in the study.

### Data Collection and Analysis

Interviews followed a semistructured format. Questions were asked on various uses of Web-based media, which included SNSs, mobile phones, tablets, and computers. The interview topics (Table 1) were developed by LLR with input from a sociologist.

Interviews lasted between 40 and 120 minutes, were audiorecorded, and later transcribed by a professional transcription agency. All transcripts were stored on a secured server and, when printed, in a locked filing cabinet where only numeric identifiers were used to identify participants.

Interviews were conducted by a postdoctoral researcher (RR) between May and October 2015. By way of preparation, the researcher attended a self-management course for PWE [27] to gain an understanding of epilepsy-related challenges (ie, stigma and driving restrictions). Interviews took place either in participants’ homes or a public place.

**Table 1:** Question guide.

Interview Topic	Questions
Diagnosis	What kind of epilepsy do you have? When were you diagnosed?
General experience of epilepsy offline	Do you keep a symptoms or medication diary? Reminders? Family member? Do you talk to people about your epilepsy? Who? Do you know anyone else with epilepsy? Fictional characters? Have you ever been to a support group? What was it like? Charity work? Advocacy? Why did you go?
General use of the internet and social media	What sites do you use? Twitter, Facebook, Email, Tumblr, Blogs, Instagram, LinkedIn, Forums, or chat rooms? How often? When did you start? Why? Have you stopped using any? Why? What devices do you use to access these sites or apps? Do you change the way you present yourself on different sites or apps? Why? Do you try to? Why?
Broad experience of epilepsy online	Do you talk about your epilepsy on these sites? In private messages? Why? Is privacy an issue? How do you think epilepsy is portrayed in general online? For example, trolling, surgery games, “seizure” on Twitter, Photosensitive-provoking gifs. Do you think your epilepsy affects the way you use these sites?
Epilepsy-specific websites	How often do you use the Epilepsy Action forum? Why? Is it different from talking offline? Do you use any other epilepsy forums or websites? Blogs? Why?—Information, friendship, advocacy? Do you think these experiences have changed the way you think about your epilepsy? Yourself? The online world? Has it changed the way you interact with doctors? Friends? Other people with epilepsy? How could these websites be improved?

We used the thematic analysis when examining interview transcripts [28]. Thematic analysis is not tied to a specific methodological orientation, making it a flexible analytic method, suitable for health services research. The lead author (ARM) read each transcript twice and drafted a document of initial impressions, which was then reviewed by the second author (LLR). A set of codes were inductively produced. The codes were then organized into an set of themes. The lead author presented and gained feedback on the initial themes to 2 groups of researchers. One group had expertise in life writing and online identity, and another had expertise in neuroscience research. The themes and codes were further refined in light of this feedback. Finally, a series of definitions were developed for each theme and the final results report produced.

## Results

### Participants’ Characteristics

There were 14 participants of an equal number of men and women (Table 2), with a median age of 50 (range: 33-73) years and the median number of 25 years since diagnosis (range: 13-63 years). Participants self-reported generalized tonic-clonic seizures (n=7), complex partial seizures (n=4), photosensitivity (n=4), and absences (n=3). Three participants disclosed a comorbid health condition, including diabetes, asthma, and physical disability.

### Silent Observation of Others on Social Media and Websites

Nine participants described using Web-based media to learn from the shared accounts and posts from other PWE. Many described reading but not necessarily posting or interacting with others; this practice was explained as a desire to keep up-to-date with epilepsy-related information, without feeling the need to respond or contribute themselves.

I have used [user-group websites] for research on epilepsy medication. That’s probably about it. I use forums for that, but I would never post on them.

p13

### Social Comparison Assisting With Epilepsy Management

Seven participants spoke about using Web-based media for social support and to find narratives from other PWE as a way of learning about their condition. The use of social comparison in this context seemed to be an indirect form of social support. One participant (p11) was able to read how other PWE had coped with the same kind of neurosurgery they were planning to have. Another participant (p13) found an explanation for the medication side effects they had experienced from taking Keppra (generic name levetiracetam, an antiepileptic drug introduced in the United States in 2008 and the United Kingdom in 2011).

[I look for] personal stories of how they’ve got on with their medication, how they’ve got on with reactions to it and that kind of thing...I found myself getting angry, so I was finding out about the whole Keppra rage thing and seeing whether other people got it.

p13

**Table 2:** The demographic characteristics of participants.

Participant number	Sex	Age	Diagnosis length (years)	Social media platforms used
p1	Female	46	43	FB^a^, Twitter, LinkedIn, user-group^b^ forum
p2	Male	35	17	FB, FB messenger, Twitter, LinkedIn
p3	Female	50	15	LinkedIn
p4	Female	38	25	FB, Twitter, LinkedIn, user-group chat rooms
p5	Male	59	36	FB
p6	Female	43	25	Facetime, FB (including private epilepsy-related FB group)
p7	Female	43	24	FB, Twitter
p8	Male	56	35	FB (including messenger), Twitter, Instagram, foursquare, WhatsApp, Snapchat, Blackberry messenger, Skype
p9	Female	63	15	Twitter
p10	Male	52	47	FB, Twitter, private message on Epilepsy Action forum
p11	Male	65	Not specified	FB, user-group chat rooms, Skype
p12	Female	64	63	FB, user-group forums
p13	Male	33	13	FB, WhatsApp, Twitter
p14	Male	73	58	FB (including messenger), Twitter

^a^FB: Facebook.

^b^UK-based charity called Epilepsy Action.

### Self-Identifying With Epilepsy

Six participants described the way they present themselves as being inseparable on websites and in real life. “Me” and “my epilepsy” were expressed as one singular identity.

I am what I am, if people don’t like me, I don’t care whether they do.

p7

One participant (p12) explained this in terms of wanting to be “as honest as possible” online and offline. One participant’s (p5) openness about epilepsy came about after observing other people disclosing their epilepsy on a nonepilepsy-related website.

### Self-Disclosure Dependent on the Audience

Five participants spoke about modifying their Web-based self-presentation depending on which SNS platform they were using, or whom they felt the audience was likely to be. When posting on a user-group chat room, as opposed to public epilepsy-related SNS pages, one participant felt he could talk more openly.

It changes the actual thing of what you want to say to the person. What they want to say to you, and obviously it makes a difference in the conversation and what it builds up to...In the epilepsy chat room, you can talk about more or less everything that goes on as on a day-to-day basis, your medication and everything...On Facebook [public epilepsy pages] you have to be careful what you are saying and what you put down.

p10

One of these participants (p7) said she did not talk about epilepsy online owing to concerns about privacy. Another (p3) was concerned about future employers learning through LinkedIn that she had left her previous job because of having a serious seizure. Several did not want the “world at large” to know about their epilepsy and, therefore, tended to only share with close friends privately or on closed epilepsy-specific Facebook groups.

### Cautious About Web-Based Disclosure and Self-Presentation

Four participants described some level of apprehension about having epilepsy-related content on their personal social media pages, as though there was a separation of their “public” Web-based identity and epilepsy-related identity. Unless directly asked by others, they did not discuss the day-to-day elements of their condition openly.

So that’s the only thing with Facebook, it highlights the good bits of what people do. It doesn’t tend to show them at their worst. I think nobody would want to be my friend if I showed my worst.

p6

On the other hand, another participant (p1) had initially felt apprehension about identity and self-disclosure offline but had since become more confident in both domains after talking about epilepsy online.

I didn’t make it public, but I didn’t entirely hide it...I would tell them, but if they didn’t ask, I wouldn’t tell them...I’m only recently trying to be more open about my epilepsy online because, for years, I wasn’t very public about it...It’s a new thing for me to able to discuss it and, for so long, I couldn’t.

p1

### Presenting Epilepsy to Web-Based Social Contacts

Four participants used Web-based media to indirectly share or create awareness about the topic of epilepsy with their social contacts. One of these participants (p1) described not feeling completely comfortable when talking about herself as someone with epilepsy but was happy to have her image on Facebook and a user-group website having a telemetry assessment. Two others described sharing images of themselves to their wider SNS networks (ie, through Twitter and Facebook) supporting epilepsy-related charities, while not openly disclosing that they have epilepsy.

The photo of me on there is the day I did my Epilepsy Action Run...I had the t-shirt on and I had my kids sat either side of me. So, you’d probably know that I had some connection to epilepsy that way.

p2

One participant spoke early in his interview of there being a shift from feeling concerned about self-disclosure of his epilepsy, to no longer being worried about the audience; this occurred once his employment situation changed, from working for someone else to running his own business:

I don’t wear a little badge saying I’m epileptic...I put it on my Facebook to make it clear to people who support it. So it doesn’t worry me now. Certainly, with the fact that I’m working for myself it doesn’t worry me.

p11

## Discussion

### Principal Findings

This study demonstrates that PWE received personally significant support and value from their SNSs and Web-based media use. For some, Web-based media was used as a forum to overcome a stigmatized identity, as well as develop connections with other PWE.

As with previous research on the uses of digital technology [25,29], PWE in this study used Web-based media to access social support. Connecting with others online about the shared experience of a long-term condition can reduce distress and provide reassurance [21]. Such benefits are particularly helpful for people with recurrent seizures, where high levels of anxiety and low quality of life are commonly reported [30].

In addition, accessing support through SNSs and user-group websites can have additional advantages over traditional face-to-face interactions. Users can read highly personal accounts from other users in several clicks; while individuals may not be as forthcoming with personal information when conversing with new social contacts offline [31]. The perceived benefit of Web-based media used in this way is the constancy of support it provides. Posts in a Facebook group are available in an asynchronous format (ie, not real time but when a user consciously checks), while private direct messages and chat provide synchronous connections with others in real time [6]. SNSs are available 24 hours a day on mobile phone or computer; thus, PWE have the potential to be connected with continual support and social connection [14]. As many PWE experience difficulties with transport, and can have unpredictable seizures, the function of Web-based media as a support group can be especially valuable.

Almost half of the participants in our group said that they modified their Web-based self-presentation depending on the perceived or *imagined audience* [32]. While in face-to-face interactions, people may perceive the audience as those whom they can see, in Web-based spaces, the audience and privacy level can vary greatly [32]. With the advent of screenshots and Web-based archiving, content posted online may be viewed by those other than whom the poster intended at the time of disclosing their personal information [33]; this creates the potential for digital stigma, where discrimination or embarrassment results from private information being publicly posted and accessed [34].

Closed Facebook groups require further evaluation as a possible tool for chronic illness management [35]. Our results show there was more of a tendency to discuss epilepsy freely in a closed epilepsy group on Facebook or message (which is considered “more private”), compared with a public Facebook page with open privacy settings. This behavior might be conceptualized in terms of theory on felt stigma, previously associated with epilepsy [36]. The perception of being different or unaccepted by others (ie, “felt stigma”) [37] may explain why some participants felt they could only share their epilepsy experiences in some areas of SNSs (ie, closed Facebook group) as opposed to others who shared openly in all settings. Modifying SNSs to address stigma-related barriers may help to improve the other benefits PWE gain from using Web-based media to self-manage their condition.

Participants reported the use of Web-based media that would be expected of individuals with general health concerns (ie, information seeking). However, the experience of epilepsy is long-term, and symptoms can be debilitating, which adds further complexity to how individuals manage their day-to-day life. PWE can experience the perception of having personal characteristics that are discrediting, a kind of “spoiled identity” [7]. As a consequence, individuals living with chronic health conditions like epilepsy can withdraw and isolate themselves from others; this creates a vicious cycle, whereby individuals do not receive support or feedback and do not learn about managing their condition. SNSs are uniquely placed to disrupt this loop and overcome a stigmatized or spoiled identity by providing feedback from other PWE, observing others online, while maintaining anonymity. Over time, this may translate into changes in individual self-presentation as perceptions of identity are modified.

Changes in self-presentation over time were described by several participants, through either modeling observed disclosures of other PWE or using Web-based media to improve self-confidence. One participant, in particular, felt more able to talk about her epilepsy offline after discussing her condition with others online. This could be explained as a form of *identity play*, whereby profound development and personal change are precipitated by the life one leads using Web-based media [38]. For this participant, her interactions online provided a place to monitor or experiment with epilepsy-related identity performance, which affected her offline life. This form of identity play or management has been identified with other patient groups using Web-based spaces, but with the added consideration that if privacy is compromised throughout this process, this could cause great distress to an individual [20].

In this study, almost half of the participants said that their self-presentation was the same regardless of the Web-based platform. They were also more likely to be “openly out” about their epilepsy in their offline lives. These SNS users may perceive a kind of social currency with portraying their authentic selves as a way of developing connections or engaging others online [32]. Several participants carried on long-distance friendships with PWE they had initially spoken with, which could further explain personal motivations to self-present in a way they felt was congruent with their actual selves [39]. The possibility of meeting face-to-face may also increase the likelihood of SNS users presenting their actual rather than idealized selves [40].

### Study Implications

Findings from this study demonstrate how a group of PWE used Web-based media to manage elements of their epilepsy, through silent observation and making connections with others. In addition, Web-based media can facilitate a dialogue between health care professionals and patients. By reading shared Web-based experiences, health care professionals may gain insight into patient responses after using their services [20]. Some suggest health care practitioners may further support PWE by increasing their Web-based presence and engaging in public discussion about epilepsy care [41]. Despite concerns about Web-based information accuracy, evidence from research into other health care conditions shows forum posts of this nature are likely to be accurate [8] and, therefore, valuable to PWE; however, professional moderation and fact-checking is necessary [21].

Many PWE reported looking at social media (ie, forums and Facebook groups) for epilepsy-related information but not interacting with other users; this is also an important finding for health care providers who post educational content online, as PWE may gain value from epilepsy-related information without necessarily interacting or participating. Social media is inexpensive to use and may be an effective means of delivering health care information to the public [42]. However, without safety protocols in place, this can restrict the development of Web-based media as a tool for health care purposes [43]. Further training and support for those using these platforms for health care purposes would be needed [5,44].

In the past decade, issues of user privacy and data use have become linked to SNSs and can affect how users present themselves online [45]. An awareness of these issues can influence an individual’s propensity to self-disclose personal information online [20,46]. While many of our participants were willing to share their experiences and seemed to find value in sharing with other PWE, many were also concerned about privacy or oversharing information on public SNSs. The benefits of mutual self-disclosure and information sharing may be enhanced if SNS platforms offer functions that do more to protect the privacy and confidentiality of users [10]. Closed Facebook groups, private messages, and user-group forums seemed to provide such a function for PWE in this study.

### Strengths and Limitations

To the best of our knowledge, this was the first study of its kind to include the perspective of PWE on their use of Web-based media relating to their identity and self-presentation. Although, in seeking to describe how PWE use Web-based media to understand and self-manage their epilepsy, the study has several limitations. The people in our study were volunteers recruited from a user-group charity and identified by their use of Web-based media most broadly. Our participants were at various tech levels, ranging from early adopters who have greater digital literacy to those who use the internet and social media infrequently; they were more likely to be aged in their forties and above and had been living with epilepsy for >25 years. Future research with participants earlier on in their illness trajectory may help identify additional themes not captured in this study. It is also likely that adults aged 18-29 years, who are more frequent users of SNSs [47], would offer a different perspective, such as the potential risks and consequences of Web-based media, than those described in this study. These demographic characteristics are, nevertheless, comparable to participants of National Health Service users, recruited into a trial of group education for PWE in England [30].

### Conclusions

Communication facilitated through digital technology and Web-based media has the potential to powerfully reshape an individual’s experience of living with a long-term condition [9]. Self-presentation and Web-based disclosure are complex processes, reflecting the challenges posed by stigmatized identities. Although Web-based identity may appear to be represented through “static” posts or tweets, it is a constant reconstructive and reflexive process [15], with the potential to be impacted by affiliation with health-related charities and causes, as was described by several in this study. The use of SNSs to gather information and support represents an opportunity for health care providers and researchers to utilize SNS platforms to improve the patient experience. Some participants changed their self-presentation for reasons such as employment consequences, with a fear of stigma modifying their SNS use. Learning to guard, present, and manage their image relationally was an important benefit of using Web-based media for people in coming to terms with the stigma of epilepsy.

## Abbreviations

PWE: people with epilepsy
SNS: social networking site
UK: United Kingdom
